# Infrared Low-Level Laser Therapy (Photobiomodulation Therapy) before Intense Progressive Running Test of High-Level Soccer Players: Effects on Functional, Muscle Damage, Inflammatory, and Oxidative Stress Markers—A Randomized Controlled Trial

**DOI:** 10.1155/2019/6239058

**Published:** 2019-11-16

**Authors:** Shaiane Silva Tomazoni, Caroline dos Santos Monteiro Machado, Thiago De Marchi, Heliodora Leão Casalechi, Jan Magnus Bjordal, Paulo de Tarso Camillo de Carvalho, Ernesto Cesar Pinto Leal-Junior

**Affiliations:** ^1^Physiotherapy Research Group, Department of Global Public Health and Primary Care, University of Bergen, Bergen, Norway; ^2^Laboratory of Phototherapy and Innovative Technologies in Health (LaPIT), Nove de Julho University, São Paulo, SP, Brazil; ^3^Postgraduate Program in Rehabilitation Sciences, Nove de Julho University, São Paulo, SP, Brazil; ^4^Faculty Cenecista of Bento Gonçalves (CNEC), Bento Gonçalves, RS, Brazil

## Abstract

The effects of preexercise photobiomodulation therapy (PBMT) to enhance performance, accelerate recovery, and attenuate exercise-induced oxidative stress were still not fully investigated, especially in high-level athletes. The aim of this study was to evaluate the effects of PBMT (using infrared low-level laser therapy) applied before a progressive running test on functional aspects, muscle damage, and inflammatory and oxidative stress markers in high-level soccer players. A randomized, triple-blind, placebo-controlled crossover trial was performed. Twenty-two high-level male soccer players from the same team were recruited and treated with active PBMT and placebo. The order of interventions was randomized. Immediately after the application of active PBMT or placebo, the volunteers performed a standardized high-intensity progressive running test (ergospirometry test) until exhaustion. We analyzed rates of oxygen uptake (VO_2 max_), time until exhaustion, and aerobic and anaerobic threshold during the intense progressive running test. Creatine kinase (CK) and lactate dehydrogenase (LDH) activities, levels of interleukin-1*β* (IL-1-*β*), interleukin-6 (IL-6), and tumor necrosis factor alpha (TNF-*α*), levels of thiobarbituric acid (TBARS) and carbonylated proteins, and catalase (CAT) and superoxide dismutase (SOD) activities were measured before and five minutes after the end of the test. PBMT increased the VO_2 max_ (both relative and absolute values—*p* < 0.0467 and *p* < 0.0013, respectively), time until exhaustion (*p* < 0.0043), time (*p* < 0.0007) and volume (*p* < 0.0355) in which anaerobic threshold happened, and volume in which aerobic threshold happened (*p* < 0.0068). Moreover, PBMT decreased CK (*p* < 0.0001) and LDH (*p* < 0.0001) activities. Regarding the cytokines, PBMT decreased only IL-6 (*p* < 0.0001). Finally, PBMT decreased TBARS (*p* < 0.0001) and carbonylated protein levels (*p* < 0.01) and increased SOD (*p* < 0.0001)and CAT (*p* < 0.0001) activities. The findings of this study demonstrate that preexercise PBMT acts on different functional aspects and biochemical markers. Moreover, preexercise PBMT seems to play an important antioxidant effect, decreasing exercise-induced oxidative stress and consequently enhancing athletic performance and improving postexercise recovery. This trial is registered with Clinicaltrials.gov NCT03803956.

## 1. Introduction

In soccer, as well as in other high-level sport activities, players experience acute muscle fatigue in the hours or even days following a single match [1, 2]. Since the number of competitive matches per season is very high (up to 65 to 76 matches per season) and the time to recovery between two successive matches may be insufficient (around 72 to 96 hours), players may experience also chronic fatigue [1–3]. The development of muscle fatigue is a complex and multifaceted process that may be associated with muscular oxidative stress [4] caused by increased reactive oxygen species (ROS) production after strenuous exercise [5, 6]. The process of fatigue leads to a decline in physical performance and injury in some cases [1–3]; therefore, it is indispensable that athlete recovery be as fast and effective as possible [7].

A recovery strategy involves the implementation of a single technique or a combination of techniques in order to enhance and accelerate recovery after matches and to best prepare the athlete for the next match, besides potentially reducing the risk of injuries [7, 8]. Among them, the most widely used are nutritional strategies [9–12], active recovery [13, 14], cold water immersion [15, 16], compression garments [17], and massage [18]. However, the evidence about the effectiveness of some of these strategies to recovery is still conflicting [17–19].

Some ergogenic agents associated with recovery strategies may be used for enhanced performance in high-level sports activities [20]. Supplements such as creatine are widely used for this purpose [21]. Currently, increasing evidence has demonstrated the potential of photobiomodulation therapy (PBMT) as an ergogenic agent, since it is able to enhance athletic performance, as well as improve postexercise recovery [22–29].

PBMT is a nonthermal therapy that uses nonionizing light sources, such as lasers, light-emitting diodes (LEDs), and broadband light from the visible to the infrared spectrum, to interact with chromophores and trigger photochemical and photophysical reactions in different tissues [30]. Studies have demonstrated the positive effects of PBMT in improving biomarkers related to muscle damage [31], modulating inflammation [32, 33], and decreasing oxidative stress [33, 34]. Moreover, it has been shown that PBMT is able to increase the number of repetitions, time until exhaustion, and peak torque (strength) if applied before an exercise session [31]. Despite the existence of data reporting neutral effects of PBMT on athletic performance, this absence of benefit is often related to the lack of adherence to the optimal or optimized parameters and/or to the clinical and scientific recommendations to apply the therapy [30, 31]. Moreover, the current evidence points to beneficial effects of this therapy (for more details, read the clinical and scientific recommendations authored by Leal-Junior et al. [30] and the systematic review with meta-analysis authored by Vanin et al. [31]).

In recent years, there was an exponential increase of evidence about the use of PBMT for enhancing performance and also accelerating postexercise recovery. However, some aspects have not been fully and/or well established so far. There are still few studies that have been performed in order to investigate the effects of PBMT as an ergogenic agent in high-level athletes [7, 32, 33, 35–37], and studies on the effects of PBMT on aspects related to aerobic endurance, for instance, are even more scarce [36]. In addition, although some studies have investigated the effects of preexercise PBMT on oxidative stress [33–35], the evidence remains conflicting, and the role of the therapy as redox intervention to enhance performance and postexercise recovery is still not well understood.

To the best of our knowledge, there are no studies evaluating the acute effects of PBMT on aerobic capacity, as well as on markers of muscle damage, inflammation, and oxidative stress of high-level soccer players. Moreover, differently to another previous study investigating the effects of infrared low-level laser therapy PBMT in a progressive running test in healthy untrained individuals [34], our study used an optimized dose [32] and optimized power output [33] previously tested in high-level soccer athletes.

Then, we hypothesized that preexercise PBMT, with optimized parameters, can increase aerobic capacity and decrease muscle damage and inflammation through decreased oxidative stress and increased antioxidant activity.

Therefore, we performed this study aiming to evaluate the effects of PBMT (using infrared low-level laser therapy) applied before a progressive running test on functional aspects, muscle damage, and inflammatory and oxidative stress markers in high-level soccer players.

## 2. Methods

### 2.1. Study Design and Ethics Statements

A randomized, triple-blind (evaluators, therapist, and volunteers), placebo-controlled crossover trial was performed at the Laboratory of Phototherapy and Innovative Technologies in Health (LaPIT), Nove de Julho University, São Paulo, Brazil. This trial received approval from the institutional Research Ethics Committee (Protocol No. 397774/2011), and the protocol has been prospectively registered on Clinicaltrials.gov (NCT03803956). There were no deviations from the registered protocol.

### 2.2. Characterization of Sample

Twenty-two high-level male soccer players from the same team were recruited. The sample size was calculated based on a previous trial conducted by our research group [34], in which the primary outcome (oxygen uptake relative to body mass—VO_2 max_ relative), the PBMT device, and the standardized progressive running exercise protocol were the same employed in our study. The sample size was calculated considering a *β* of 20% and an *α* of 5%. The decision to recruit volunteers from the same team was made to enhance the homogeneity of the sample.

### 2.3. Inclusion Criteria

- High-level soccer players
- Age between 18 and 35 years
- Male gender
- Minimum of 80% participation in team practice sessions in the last two months
- Agreement to participate through signed statement of informed consent

### 2.4. Exclusion Criteria

- History of musculoskeletal injury to hips, knees, or ankles in the previous 2 months
- Use of pharmacological agents for chronic injuries/diseases
- Smokers and alcoholics
- Occurrence of musculoskeletal injury during the study
- Any change in practice routine in relation to the rest of the team during the study
- Any change in nutritional routine (including supplements intake) during the study

### 2.5. Randomization and Blinded Procedures

The order of intervention was randomized. We generated codes through a website (random.org) to ensure that 50% of the volunteers received the active intervention and 50% of the volunteers received the placebo intervention at the first stage, in order to counterbalance participants between the two interventions tested (active PBMT and placebo PBMT) during execution of the two stages. During the second stage, volunteers received the opposite treatment compared to their first stage. Randomization was performed by a participating researcher not involved with the recruitment or evaluation of volunteers. This same researcher was responsible for programming the PBMT device according to the result of the randomization and he/she was instructed not to disclose the programmed intervention to the therapist, the assessors, or any of the volunteers and other researchers involved in the study until its completion. The PBMT device used in the present study emitted the same sounds, regardless of the programmed mode (active or placebo PBMT), and the infrared light was not visible. Concealed allocation was achieved through the use of sequentially numbered, sealed, and opaque envelopes.

### 2.6. Interventions

The active PBMT and placebo PBMT were performed using the same device and the irradiated sites were the same in both therapies.

#### 2.6.1. Photobiomodulation Therapy

**Figure 1:**
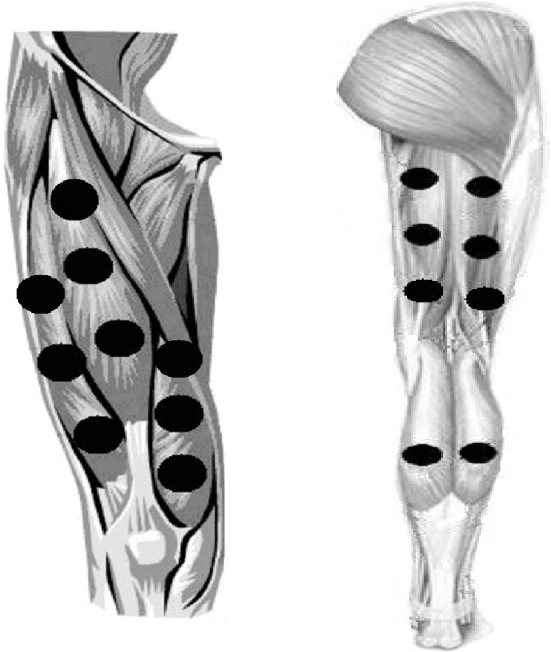
PBMT irradiation sites in the anterior and posterior regions of lower limbs.

A five-diode cluster laser device was used. The optical power was verified before irradiation in each volunteer using a Thorlabs thermal power meter (Model S322C; Thorlabs, Newton, NJ, USA). PBMT was applied using a dose of 10 J per diode (50 J per site) as previously optimized by Aver Vanin et al. [32], and with a power output of 100 mW also previously optimized by De Oliveira et al. [33]. The full description of PBMT parameters is provided in Table 1. PBMT was performed in direct contact with the skin at nine different sites of the knee extensor muscles (three medial, three lateral, and three central sites), six different sites of the knee flexor muscles (three medial and three lateral sites), and two different sites of the ankle plantar flexor muscles (one medial and one lateral site) of both lower limbs, and these sites were the same used in previous studies [7, 38, 39] (Figure 1). As the cluster had 5 diodes and 17 different sites were irradiated, a total of 85 points were irradiated in each lower limb, with a total of 850 J of energy delivered per lower limb (50 J per site). The use of a cluster probe was fundamental, considering the extensive irradiation area covered in the present study.

#### 2.6.2. Placebo Therapy

Placebo PBMT was delivered using the same device as the active PBMT but without any emission of therapeutic dose or power. Volunteers received a total dose of 0 J in the placebo mode. Placebo PBMT was applied in the same sites and with the same time of irradiation as the active PBMT. To ensure blinding, the device emitted the same sound regardless of the programmed mode (active or placebo PBMT).

### 2.7. Procedures

**Figure 2:**
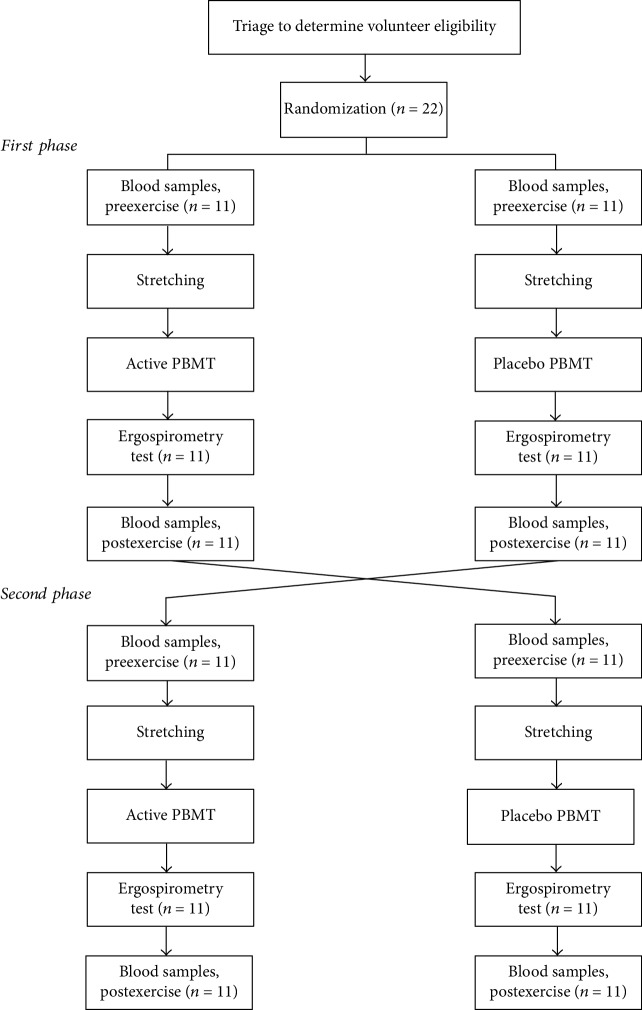
Flowchart of the study.

The study was performed in two stages, since it was a crossover study, with a washout period of 14 days between stages. The Consolidated Standards of Reporting Trials flowchart that summarizes the experimental procedures and volunteers is shown in Figure 2.

#### 2.7.1. Blood Samples and Biochemical Analysis

Blood samples (10 ml) were taken by a qualified nurse (blinded to group allocation) from the antecubital vein of the volunteers before the stretching and intervention (active or placebo PBMT) and then, exactly 5 minutes after the intense progressive running test (ergospirometry test), blood samples were collected again. Up to one hour after collection, each sample was centrifuged at 3000 rpm for 20 min. Pipettes were used to transfer the serum to Eppendorf tubes, which were stored at -80°C until analysis. Blood analysis involved the determination of specific skeletal muscle creatine kinase (CK) isoform and lactate dehydrogenase (LDH) activities through spectrophotometry and specific reagent kits (Labtest, Brazil) following the manufacturer's instructions. The analyses of interleukin-1*β* (IL-1*β*), interleukin-6 (IL-6), and tumor necrosis factor alpha (TNF-*α*) levels were performed employing enzyme-linked immunosorbent assays (ELISA) and specific reagents (BD Biosciences, USA). The analyses of thiobarbituric acid (TBARS), carbonylated proteins, catalase (CAT), and superoxidedismutase (SOD) activities were performed using spectrophotometry and specific reactions previously described in literature [40–43].

#### 2.7.2. Stretching

Immediately after the collection of blood samples, the volunteers performed one 60 sec set of active stretching exercise of the knee extensors and flexors; hip extensors, flexors, abductors, and adductors; and ankle plantar and dorsal flexor muscles of both lower limbs.

#### 2.7.3. Application of Therapy

Immediately after performing stretching, the volunteers received an application of active PBMT or placebo PBMT according to prior randomization.

#### 2.7.4. Intense Progressive Running Test (Ergospirometry Test)

Immediately after the application of intervention (active or placebo PBMT), skin asepsis of the volunteers and electrode placement (for cardiac monitoring) were performed. Then the nozzle coupled to the gas analyzer was placed in volunteers, as well as the nasal clip. The ergospirometry test started exactly 5 minutes after the application of the interventions (active or placebo PBMT). We used a progressive treadmill exercise protocol, previously used in studies conducted by our research group [34, 38, 39]. Volunteers performed a standardized progressive running protocol on a motor-driven treadmill with a fixed inclination of 1%. The initial velocity was 3 km/h during the first 3 minutes (warm-up phase). After the warm-up phase, the velocity was increased 1 km/h at each minute until it reached 16 km/h. Volunteers performed the exercise protocol until exhaustion, and they were instructed to use hand signals to request termination of the test at any time. After the exercise protocol, the volunteers performed a recovery phase with a velocity of 6 km/h. The entire test was monitored by electrocardiogram and blood pressure measurements. The test was stopped if any abnormal changes were observed in heart rate or blood pressure by the assessor in charge of the test, or if the test was terminated prematurely on request by the volunteer.

### 2.8. Outcomes

The primary outcome was oxygen uptake relative to body mass (VO_2 max_ relative) measured by the software of the ergospirometry system, during the intense progressive running test. The secondary outcomes were rates of VO_2 max_ in absolute values, time until exhaustion, and aerobic and anaerobic threshold, measured through the ergospirometer during the intense progressive running test. Moreover, muscle damage (CK and LDH), inflammation (IL-1*β*, IL-6, and TNF-*α*), and oxidative stress (TBARS, carbonylated proteins, CAT, and SOD) were measured through blood samples collected before and 5 minutes after the end of the intense progressive running test. All assessments were performed by a blinded researcher.

### 2.9. Statistical Analysis

The statistical analysis was conducted following the principles of intention-to-treat analysis [44]. The Shapiro-Wilk test was used to determine the distribution of the data, which were then expressed as mean and standard deviation. Data regarding the ergospirometry test (rates of oxygen uptake (VO_2 max_), time until exhaustion, and aerobic and anaerobic thresholds) were analyzed using the paired, two-tailed Student *t*-test. Data regarding biochemical analysis were analyzed using a two-way ANOVA test, followed by the Bonferroni post hoc test. The significance level was set at *p* < 0.05. Data in graphs are expressed as mean and standard error of the mean (SEM).

## 3. Results

Twenty-two male high-level soccer players from the same team were recruited and finished all the procedures of this study. Therefore, there were no dropouts and the intention-to-treat analysis was not necessary. The mean age of volunteers was 18.85 (±0.61), height was 175.84 cm (±4.01), and body mass was 68.22 kg (±8.26). No adverse effects were reported during the study.

In order to assess possible residual effects of PBMT and placebo from the first test to the second test, we performed an analysis of variables regarding the progressive running test, and also baseline values of all biochemical markers. This analysis was performed only considering the values obtained in test 1 and test 2, regardless the treatment given before the tests. As can be observed in Table 2, there were no residual effects.

**Figure 3:**
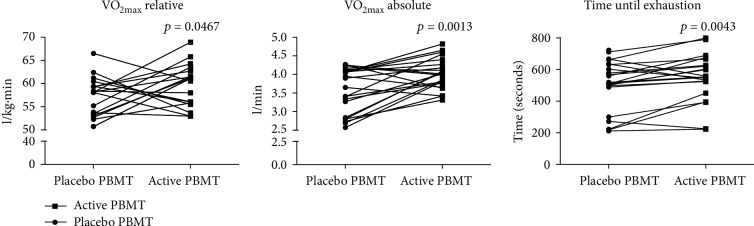
Oxygen uptake—VO_2 max_ (relative and absolute)—and time until exhaustion.

The application of active PBMT before the intense progressive running test significantly increased the oxygen uptake (VO_2 max_), both in relative (*p* < 0.0467) and absolute values (*p* < 0.0013), as well as the total time until exhaustion (*p* < 0.0043) compared to the application of preexercise placebo PBMT (Figure 3). Moreover, active PBMT significantly increased the anaerobic threshold, both in time (*p* < 0.0007) and volume (*p* < 0.0355), while it only significantly increased aerobic threshold in volume (*p* < 0.0068), compared to placebo PBMT.

**Figure 4:**
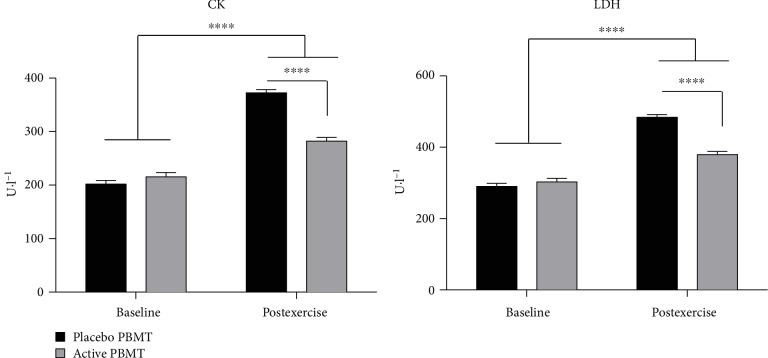
Activity of CK and LDH. Data are expressed as mean and SEM. ^∗∗∗∗^*p* < 0.0001.

Regarding assessment of muscle damage, the results demonstrated that active PBMT applied before the exercise protocol significantly decreased the activity of both postexercise CK and LDH (*p* < 0.0001) compared to placebo PBMT (Figure 4).

**Figure 5:**
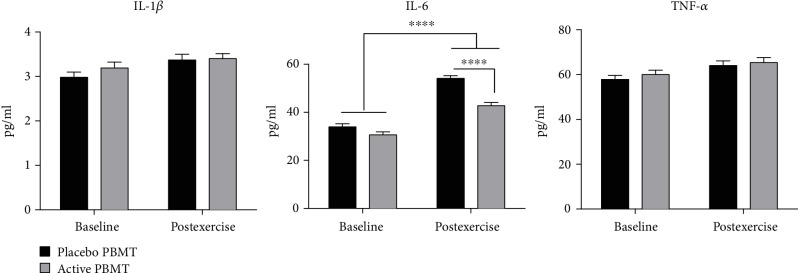
Levels of IL-1*β*, IL-6, and TNF-*α*. Data are expressed as mean and SEM. ^∗∗∗∗^*p* < 0.0001.

The evaluation of inflammatory markers demonstrated that there was no significant difference between active PBMT and placebo PBMT, applied before the exercise protocol, on postexercise levels of IL-1*β* and TNF-*α*. However, active PBMT decreased significantly the postexercise levels of IL-6 (*p* < 0.0001) when compared to placebo PBMT (Figure 5).

**Figure 6:**
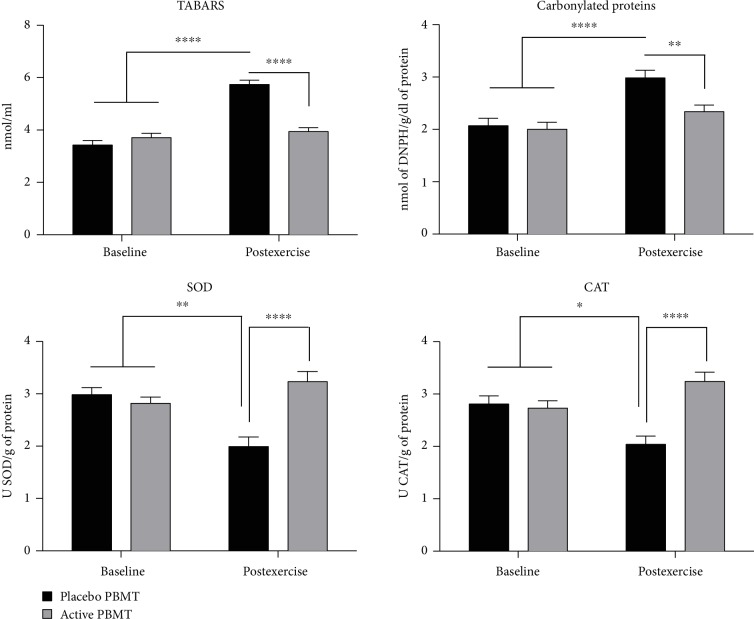
Levels of TBARS and carbonylated proteins and activity of SOD and CAT. Data are expressed as mean and SEM. ^∗^*p* < 0.05, ^∗∗^*p* < 0.01, and ^∗∗∗∗^*p* < 0.0001.

For oxidative stress markers, active PBMT applied before the exercise protocol significantly decreased postexercise TBARS (*p* < 0.0001) and carbonylated protein levels (*p* < 0.01) when compared to placebo PBMT. Moreover, active PBMT significantly increased SOD (*p* < 0.0001) and CAT (*p* < 0.0001) postexercise activity compared to placebo PBMT (Figure 6). All the outcomes (mean and standard deviation) about the functional aspects, muscle damage, and inflammatory and oxidative stress markers are fully described in Table 3.

## 4. Discussion

This study evaluated for the very first time the effects of PBMT (using infrared low-level laser therapy with optimized parameters) applied before a progressive running test on aerobic endurance, muscle damage, inflammatory process, and oxidative stress of high-level soccer athletes. In general, our results demonstrated that PBMT applied before an aerobic exercise protocol is effective in improving functional and biochemical aspects, enhancing athletic performance and postexercise recovery. Differently to another previous study investigating the effects of infrared low-level laser therapy PBMT in a progressive running test in healthy untrained individuals [34], our study used an optimized dose [32] and optimized power output [33] previously tested in high-level soccer athletes.

Evidence has shown that PBMT applied before exercise is able to increase aerobic endurance in healthy, nonathletic subjects [34, 38] as well as in competitive cyclists [36]. To the best of our knowledge, our study is the first to investigate the effects of preexercise PBMT on aerobic endurance in high-level soccer athletes, and we observed that the therapy was able to increase oxygen uptake (VO_2 max_) and athletes' anaerobic and aerobic thresholds during exercise. In addition, we observed that preexercise PBMT was effective in increasing time to reach exhaustion during the intense progressive running test, corroborating with a recent systematic review, as well as with the recent clinical and scientific recommendations made by experts in this field [31]. The effect of PBMT on muscle cells, in particular increasing cytochrome c-oxidase activity [45, 46], may increase cell metabolism and ATP production. Thus, our results suggest that this mechanism of PBMT contributed to modulate aerobic endurance, and therefore, to enhance the performance of high-level soccer players, increasing both oxygen uptake and time to reach exhaustion.

Muscle damage can disturb the time course of recovery and performance of soccer athletes after matches, and currently some biomarkers, such as CK and LDH, are monitored daily to assess the muscle status of soccer athletes [3]. Several studies have demonstrated that preexercise PBMT is able to decrease both CK and LDH postexercise activities [25, 32–35] and consequently contribute to performance enhancement and postexercise recovery. Our results corroborate with studies performed with nonathletes [25, 34] and high-level athletes [24, 32, 33, 35], in which the irradiation of PBMT before the exercise was able to decrease the expected increase in these enzyme activities. On the other hand, one study also performed with soccer players showed that preexercise PBMT was not effective in decreasing CK activity [47]. However, it is important to highlight that several PBMT parameters used by Dos Reis et al. [47] were very different from those used in our study, such as dose per point, dose per site, total dose, power output, energy density, power density, and length of irradiation. This demonstrates very clearly that previous optimization of PBMT parameters is paramount for the effectiveness of the therapy as previously demonstrated by De Marchi et al. [48].

The systemic increase of muscle damage markers due to a soccer match may be responsible for triggering an inflammatory response in athletes, increasing cytokine levels, such as TNF-*α* and IL-6 [49–51]. Our results demonstrated that the preexercise PBMT was effective in decreasing IL-6 levels, as demonstrated in previous studies [32, 33]. We believe that the modulation of this cytokine has been an important aspect in the performance enhancement and postexercise recovery of athletes, since it is one of the most potent mediators of the acute phase of inflammatory response in skeletal muscles. On the other hand, our results demonstrated that IL-1*β* and TNF-*α* levels remained unchanged. Our results corroborate with the previous study carried out by De Oliveira et al. [33], in which preexercise PBMT did not change the levels of these cytokines immediately after an eccentric exercise protocol. However, this same study [33] demonstrated that the peak release of IL-1*β* and TNF-*α* occurred between 24 and 48 hours after the exercise protocol. Despite the differences between a progressive running test and an eccentric exercise protocol, this aspect could partially explain our results, since, possibly immediately after the progressive running test there still was no increase in these cytokines levels. Therefore, further research must assess these markers between 24 and 48 hours.

Besides muscle damage, physical exercise that generates high-intensity muscle contractions leads to increased ROS production, and consequently oxidative stress [6]. Our results demonstrated that preexercise PBMT was able to decrease lipid peroxidation and carbonylated protein production, related to oxidative damage to lipids and proteins caused by exercise protocol, as previously demonstrated [33, 35]. On the other hand, our results diverged from the findings of De Marchi et al. [34], in which preexercise PBMT was not able to modulate CAT activity, reinforcing the importance of optimizing PBMT parameters to achieve positive results in all outcomes related to oxidative stress and antioxidant activity. Moreover, it is important to highlight that our study was performed with high-level athletes while the study by De Marchi et al. [34] was performed with nonathletes, which could also partially explain the difference of results between studies. Furthermore, also in disagreement with our results, there was evidence that preexercise PBMT was not able to modulate SOD and CAT activity [33]. To date, the evidence regarding the effects of PBMT on exercise-induced muscular oxidative damage is sparse and divergent. Finally, our outcomes regarding oxidative stress markers and antioxidant activity might diverge from some studies [52]. However, it is important to highlight that this is a controversial aspect with conflicting findings in the literature, and therefore, further studies are needed. Further research must also investigate how long preexercise PBMT can upregulate antioxidant activity.

Our results also demonstrated that preexercise PBMT was able to modulate redox activity, increasing the activity of SOD and CAT, enzymes responsible for preventing oxidative damage. Our findings were promising to demonstrate that the therapy has a potential antioxidant effect, playing an important role in the enhancement of athletic performance and postexercise recovery. Thus, we suggest that one of the possible mechanisms of action by which PBMT is able to promote this enhancement in both performance and postexercise recovery is precisely through the upregulation of antioxidant activity and consequently decreasing exercise-induced oxidative stress. Further studies evaluating the effects of preexercise PBMT on different oxidative stress markers are needed to confirm our results and also to establish this therapy as an antioxidant agent. In addition, it is important to further investigate the possible redox mechanism promoted by PBMT.

### 4.1. Strengths and Limitations

The strengths of this study are the high methodological quality, since it is a triple-blinded, randomized, placebo-controlled and prospectively registered clinical trial. The sample size was calculated to provide the appropriate statistical power to detect a precise difference in the primary outcome. Moreover, the statistical analysis was designed following the principles of intention-to-treat analysis. Finally, the parameters of PBMT used were previously optimized in order to investigate the effects of PBMT application before an exercise protocol performed by high-level athletes, without extrapolation between populations. The limitation of the study was not having measured the effects of PBMT on local oxygen concentration (oxyhemoglobin, deoxyhemoglobin, and total hemoglobin), as well as only assessing immediate effects.

## 5. Conclusion

In summary, our results demonstrated that preexercise PBMT as a stand-alone therapy was able to improve different functional aspects related to athletic performance and biochemical markers related to muscle damage and inflammatory process in high-level athletes. In addition, it is important to highlight that preexercise PBMT had an interesting antioxidant effect, being able to decrease exercise-induced oxidative stress, which suggests that this might be one of the possible mechanisms of action through which PBMT promotes ergogenic effects and protective effects to skeletal muscles. It is very likely that the sum of the different mechanisms of action was determinant for the therapy to improve aerobic endurance and postexercise recovery.

## Figures and Tables

**Table 1:** PBMT parameters and specifications.

Wavelength	810 nm (infrared)
Number of diodes	5
Frequency	Continuous output
Optical output (per diode)	100 mW or 0 mW (placebo)
Spot size (per diode)	0.0364 cm^2^
Power density (per diode)	2.75 W/cm^2^ or 0.00 W/cm^2^ (placebo)
Energy (per diode)	10 J
Energy density (per diode)	275 J/cm^2^ or 0 J/cm^2^ (placebo)
Exposure time	100 s
Number of irradiated sites per lower limb	9 sites on knee extensor muscles (3 medial, 3 lateral, and 3 central) 6 sites on knee flexor muscles (3 medial and 3 lateral) 2 sites on plantar flexor muscles (1 medial and 1 lateral)
Total number of points per lower limb	85
Total energy delivered per lower limb	850 J (450 J on knee extensor muscles, 300 J on knee flexor muscles, and 100 J on plantar flexor muscles)
Cluster area	9.6 cm^2^
Administration technique	Cluster in stationary position with slight pressure and direct contact with skin

**Table 2:** Outcomes in test 1 vs. test 2 regardless of the treatment given (values are expressed as mean and standard deviation).

		Baseline	Postexercise	*p* value
VO_2 max_ relative (l/kg·min)	Test 1 Test 2	—	58.50 (±5.22) 57.67 (±5.68)	0.62
VO_2 max_ absolute (l/min)	Test 1 Test 2	—	3.75 (±0.59) 3.81 (±0.54)	0.73
Time until exhaustion (s)	Test 1 Test 2	—	535.91 (±161.82) 532.88 (±159.59)	0.95
Anaerobic threshold (s)	Test 1 Test 2	—	415.60 (±62.16) 409.15 (±65.12)	0.74
Anaerobic threshold (l/min)	Test 1 Test 2	—	3.15 (±0.47) 3.09 (±0.51)	0.69
Aerobic threshold (s)	Test 1 Test 2	—	179.42 (±48.21) 181.78 (±46.28)	0.87
Aerobic threshold (l/min)	Test 1 Test 2	—	2.08 (±0.34) 2.14 (±0.41)	0.60
CK (U·l^−1^)	Test 1 Test 2	217.96 (±36.12) 228.66 (±38.17)	—	0.34
LDH (U·l^−1^)	Test 1 Test 2	289.81 (±45.87) 294.09 (±43.80)	—	0.75
IL-1*β* (pg/ml)	Test 1 Test 2	3.18 (±0.49) 3.11 (±0.54)	—	0.65
IL-6 (pg/ml)	Test 1 Test 2	33.65 (±5.99) 34.78 (±5.74)	—	0.53
TNF-*α* (pg/ml)	Test 1 Test 2	57.37 (±8.98) 59.39 (±8.79)	—	0.46
TBARS (nmol/ml)	Test 1 Test 2	3.90 (±0.80) 3.67 (±0.85)	—	0.36
Carbonylated proteins (nmol of DNPH/g/dl of protein)	Test 1 Test 2	2.14 (±0.59) 2.07 (±0.68)	—	0.72
SOD (U SOD/g of protein)	Test 1 Test 2	2.85 (±0.69) 2.97 (±0.72)	—	0.58
CAT (U CAT/g of protein)	Test 1 Test 2	2.82 (±0.74) 2.76 (±0.78)	—	0.79

**Table 3:** Outcomes (values are expressed as mean and standard deviation).

		Baseline	Postexercise
VO_2 max_ relative (l/kg·min)	Placebo PBMT Active PBMT	—	55.69 (±5.55) 59.40 (±5.08)^a^
VO_2 max_ absolute (l/min)	Placebo PBMT Active PBMT	—	3.57 (±0.60) 4.02 (±0.41)^a^
Time until exhaustion (s)	Placebo PBMT Active PBMT	—	504.59 (±160.44) 563.27 (±159.46)^a^
Anaerobic threshold (s)	Placebo PBMT Active PBMT	—	384.86 (±63.53) 440.73 (±58.58)^a^
Anaerobic threshold (l/min)	Placebo PBMT Active PBMT	—	2.93 (±0.46) 3.26 (±0.40)^b^
Aerobic threshold (s)	Placebo PBMT Active PBMT	—	169.73 (±45.40) 184.36 (±44.84)
Aerobic threshold (l/min)	Placebo PBMT Active PBMT	—	2.20 (±0.43) 2.53 (±0.29)^a^
CK (U·l^−1^)	Placebo PBMT Active PBMT	201.76 (±32.98) 215.61 (±37.22)	372.43 (±29.11) 282.03 (±33.26)^c^
LDH (U·l^−1^)	Placebo PBMT Active PBMT	290.02 (±41.82) 302.13 (±46.73)	483.55 (±38.09) 378.97 (±41.52)^c^
IL-1*β* (pg/ml)	Placebo PBMT Active PBMT	2.98 (±0.56) 3.19 (±0.62)	3.37 (±0.61) 3.40 (±0.54)
IL-6 (pg/ml)	Placebo PBMT Active PBMT	33.92 (±6.09) 30.6 (±5.89)	54.08 (±5.56) 42.77 (±6.01)^c^
TNF-*α* (pg/ml)	Placebo PBMT Active PBMT	57.81 (±8.75) 60.04 (±9.03)	63.99 (±9.87) 65.34 (10.71)
TBARS (nmol/ml)	Placebo PBMT Active PBMT	3.42 (±0.85) 3.71 (±0.79)	5.74 (±0.78) 3.94 (±0.71)^c^
Carbonylated proteins (nmol of DNPH/g/dl of protein)	Placebo PBMT Active PBMT	2.07 (±0.67) 2.00 (±0.63)	2.98 (±0.70) 2.34 (±0.59)^a^
SOD (U SOD/g of protein)	Placebo PBMT Active PBMT	2.98 (±0.65) 2.82 (±0.55)	1.99 (±0.86) 3.23 (±0.91)^c^
CAT (U CAT/g of protein)	Placebo PBMT Active PBMT	2.81 (±0.73) 2.73 (±0.66)	2.04 (±0.75) 3.24 (±0.83)^c^

^a^Difference of placebo PBMT (*p* < 0.01). ^b^Difference of placebo PBMT (*p* < 0.05). ^c^Difference of placebo PBMT (*p* < 0.0001).

## Data Availability

The data sets generated and analyzed during the current study are available from the corresponding author on reasonable request.
